# Digital Cognitive Behavioral Therapy for Panic Disorder and Agoraphobia: A Meta-Analytic Review of Clinical Components to Maximize Efficacy

**DOI:** 10.3390/jcm14051771

**Published:** 2025-03-06

**Authors:** Han Wool Jung, Ki Won Jang, Sangkyu Nam, Areum Kim, Junghoon Lee, Moo Eob Ahn, Sang-Kyu Lee, Yeo Jin Kim, Jae-Kyoung Shin, Daeyoung Roh

**Affiliations:** 1 Department of Psychiatry, Yongin Severance Hospital, Yonsei University College of Medicine, Yongin 16995, Republic of Korea; jhw2@naver.com (H.W.J.); kima@yuhs.ac (A.K.); 2 Mind-Neuromodulation Laboratory, Hallym University College of Medicine, Chuncheon 24253, Republic of Korea; psychang@hanmail.net (K.W.J.); skatkdrb11@hanmail.net (S.N.); bluephy@gmail.com (J.-K.S.); 3 Department of Endocrinology and Metabolism, Hallym University College of Medicine, Chuncheon 24253, Republic of Korea; brighter87@naver.com; 4 Department of Emergency Medicine, Hallym University College of Medicine, Chuncheon 24253, Republic of Korea; skyahn@hallym.or.kr; 5 Department of Psychiatry, Hallym University College of Medicine, Chuncheon 24253, Republic of Korea; skmind114@hanmail.net; 6 Department of Neurology, Kangdong Sacred Heart Hospital, Seoul 05355, Republic of Korea; yjhelena@hanmail.net; 7 Department of Psychiatry, Massachusetts General Hospital, Harvard Medical School, Boston, MA 02129, USA

**Keywords:** inhibitory learning, personalized, tailored, internet, online, therapist guide

## Abstract

**Background**: Although digital cognitive behavioral therapy (dCBT) is considered effective for anxiety disorders, there is considerable heterogeneity in its efficacy across studies, and its varied treatment content and clinical components may explain such heterogeneity. **Objective**: This review aimed to identify the efficacy of digital cognitive behavioral therapy for panic disorder and agoraphobia, and examine whether applying relevant clinical components of interoceptive exposure, inhibitory-learning-based exposure, and personalization of treatment enhances its efficacy. **Methods**: Randomized controlled trials of dCBT for panic disorder and agoraphobia with passive or active controls were identified from OVID Medline, Embase, Cochrane Library, and PsycINFO. The overall effect sizes for dCBT groups (interventions through digital platforms based on the internet, mobile, computers, VR, etc.) were aggregated against passive control (placebo/sham) and active control (traditional CBT) groups. For subgroup analysis, key intervention components such as interoceptive exposure, inhibitory learning, and personalization were assessed dichotomously (0 or 1) along with other study characteristics. The stepwise meta-regression models were applied with traditional and Bayesian statistical testing. The risk of bias and publication bias of included studies were assessed. **Results**: Among the 31 selected studies, dCBT had an overall effect size of *g* = 0.70 against passive control and *g* = −0.05 against active control. In subgroup analysis, interoceptive exposure improved the clinical effects for both controls, and inhibitory learning and personalization increased the clinical effects for passive control along with therapist guide/support and the length of sessions. Many studies were vulnerable to therapist bias and attrition bias. No publication bias was detected. **Conclusions**: The heterogeneity in clinical effects of dCBT for panic and agoraphobia can be explained by the different intervention factors they include. For effective dCBT, therapists should consider the clinical components relevant to the treatment.

## 1. Introduction

Cognitive behavioral therapy (CBT) is a well-established psychotherapy to address distorted cognition and behavior related to mental disorders. It incorporates continuous exposure to fear-related situations or stimuli and is recommended as a preferred therapy for panic disorder and agoraphobia [1]. However, its implementation in real-world settings involves significant challenges. In one instance, administering therapy to multiple patients simultaneously is difficult due to the substantial time commitment for clinical training and the concomitant increase in treatment expenses. These issues are a significant obstacle to delivering psychotherapy, as they limit patient access to CBT and finally inflate societal costs over time [2].

Aiming to overcome the limitations of conventional CBT, computer-based digital therapy has recently gained attention. Digital cognitive behavioral therapy (dCBT) employs digital platforms and technologies for treatment, including online resources or virtual reality (VR) environments [3]. Compared to conventional CBT, dCBT reduces the intensive training required for clinicians, allows for more patients with limited resources, and is economical [4]. Moreover, the outbreak of the COVID-19 pandemic resulted in an unparalleled surge in the execution of unconventional treatment delivery formats for CBT [5]. Many researchers argue that digital therapy is now rapidly reshaping the post-pandemic healthcare system, being established as a new standard in healthcare delivery [6,7].

dCBT is also recognized as an effective treatment for panic disorder and agoraphobia—common anxiety or phobic disorders characterized by sudden, marked fear and accompanying avoidance behaviors. Panic disorder is diagnosed when there are recurrent panic attacks and persistent concerns and changes in behavior tied to the attacks, and agoraphobia is diagnosed based on marked fear about public spaces or situations in which escape might be difficult or help might be unavailable [8]. Several meta-analyses suggest that the therapeutic effects of dCBT or exposure therapy are comparable to those of conventional CBT for panic disorder and agoraphobia. Fodor et al.’s (2018) meta-analysis on VR therapies for anxiety symptoms suggested a Hedges’ *g* value (between-group effect size with pooled weighted standard deviation) of 0.79 across 23 studies against passive control (placebo, sham, or waitlist groups) and Hedges’ *g* = −0.02 across 29 studies against active control, suggesting dCBT’s equivalence to conventional therapies [9]. Stech et al. (2020) concluded that the effect of dCBT for panic disorder with or without agoraphobia was *g* = 1.22 against passive control and potentially equivalent to conventional CBT based on the active control results [10].

However, these reviews have also reported substantial heterogeneity in the clinical efficacy of dCBT across their included studies. To illustrate, although many studies have reported strong or satisfactory clinical effects of dCBT, Stech et al.’s (2020) meta-analysis reported that two out of nine included studies for panic disorder, and two out of six studies for agoraphobia showed effect sizes not significantly higher than waitlist controls [10]. Various aspects may affect the inconsistent efficacy of dCBT, but one reason may be the variations in the treatment content. Although digital healthcare has led to a prolific development of therapies for various disorders, the lack of clinical direction in this process has caused disagreement about the evidence-based treatment modules required for dCBT [11]. To establish the successful content of dCBT for panic disorder and agoraphobia, an evidence base is needed regarding what clinical components can improve its effectiveness.

Previous studies have shown that differences in intervention components and modalities can explain the heterogeneity in the effects of dCBT. For instance, Pompoli et al.’s (2018) network meta-analysis on the clinical factors of various CBT programs concluded that face-to-face settings tended to have higher response rates than VR, interoceptive exposure had higher remission and response rates, and muscle relaxation tended to have low remission and response rates [12]. Therefore, the clinical components of CBT may influence its efficacy, which highlights the need to identify and understand these factors to optimize treatment outcomes.

In this review, we present three factors that affect the efficacy of dCBT for panic disorder and agoraphobia, both theoretically and empirically. The first is interoceptive exposure, which simulates the somatic sensations or stimuli per se that cause anxiety. It focuses on increasing patients’ sensitivity to their bodily sensations and the association between such sensations and anxiety or fear [13]. Interoceptive exposure is considered indispensable for treating panic and agoraphobia [1,14]. A meta-analysis indicated that for any form of CBT targeting panic, interoceptive exposure has effects that are more than 40% superior [12]. We expect that this difference would also apply to dCBT.

The second factor is intensive exposure based on the inhibitory learning principle. Craske et al. (2008, 2014) argued that in anxiety treatment, the conventional approach of aiming to immediately reduce the anxiety itself has no theoretical basis, and patients should aim to “tolerate” the anxiety for effective treatment [15,16]. Inhibitory-learning-based exposure, therefore, lets patients learn that the fear is tolerable and the feared outcomes are less likely to occur than expected [14,16]. Despite being a recently supported concept, inhibitory learning is remarkably effective for anxiety treatment. Deacon et al. (2013) reported that intensive exposure using inhibitory learning principles is more effective in conventional CBT [14]. Böhnlein et al.’s (2020) systematic review also concluded that enhancing inhibitory learning experiences can increase the success of exposure therapy for specific phobias [17]. However, no studies have identified the effect of inhibitory learning CBT in digital environments.

The third factor is personalization, which provides individually tailored modules for each patient rather than allocating the same content to everyone [18]. Recently, personalized mental health and precision medicine have become prominent areas of interest [19,20]. Moreover, this corroborates the transdiagnostic approach, a current paradigm for evidence-based psychotherapy [18,21]. Developing digital programs in which personalized or tailored content is functionally available is key to determining the success of digital therapy. Several attempts have been made to establish and verify personalized dCBTs for anxiety disorders [22,23]. Nevertheless, the clinical effect of personalized digital therapy remains unclear. Păsărelu et al.’s (2017) meta-analysis reported that although transdiagnostic or tailored dCBT had significantly greater clinical effects than disorder-specific therapy for depression and quality of life outcomes, there was no evidence of its superiority in treating anxiety symptoms [24].

To sum up, although previous reviews have demonstrated the strong efficacy of dCBT, the source of heterogeneity in effect sizes across studies has not been clearly unraveled. We expect that clinical components such as interoceptive exposure, inhibitory learning, and personalization can explain the differential efficacy across studies; these are clinically significant factors that can improve the treatment effects of dCBT, as well. Therefore, this review investigates whether the clinical efficacy of dCBT for panic disorder and agoraphobia is improved by the presence of the above three factors that may affect the efficacy of dCBT for panic disorder and agoraphobia: interoceptive exposure, inhibitory learning, and personalization. We expect that these clinical components can explain the variability in the clinical effects of dCBT for panic disorder and agoraphobia. Also, understanding the clinical role of these factors will help identify strategies needed to maximize the efficacy of dCBT.

## 2. Methods

### 2.1. Protocol

The review protocol was established based on the guidelines for systematic reviews and meta-analyses for intervention studies, developed from the National Evidence-Based Healthcare Collaborating Agency of Korea [25]. This guidance introduces standardized methodology and practical information for literature selection, risk of bias assessment, validated analytic process, and the principles of reporting based on the Cochrane Handbook and PRISMA guideline. The protocol was prepared before initiating the review, including subgroup analysis for the three tested intervention factors of interoceptive exposure, inhibitory learning, and personalization. The review and analysis were conducted without significant deviation from the protocol. The retrospectively registered protocol is available at https://osf.io/u7h8p (accessed on 28 February 2025).

### 2.2. Research Questions

This review aimed to identify the efficacy of dCBT for panic disorder and agoraphobia compared to traditional CBT or passive controls, and whether such efficacy can be improved by applying the hypothesized clinical components of interoceptive exposure, inhibitory learning, and personalization. The population (P) included patients with panic disorder or agoraphobia, and the intervention (I) included computer- or digital-based CBT, including Internet, online, or VR interventions. The comparators (C) included active (face-to-face, in vivo) and passive (waitlist, placebo/sham) controls. The outcome (O) measures were primary clinical endpoints for panic or agoraphobia symptoms.

### 2.3. Eligibility Criteria and Group Definition

The inclusion and exclusion criteria were set by standard guidelines, previous reviews, and the established research questions for this review. The included studies were (i) publications or gray literature registered in public databases in English; (ii) original studies using quantitative methods; (iii) clinical studies using behavioral interventions among human subjects; and (iv) randomized controlled trials (RCTs).

The following studies were excluded: (v) incomplete reports such as protocols/trials, with only abstracts or some parts accessible, or insufficient information for assessment; (vi) studies not primarily targeting panic disorder and agoraphobia; (vii) studies without clinical outcomes or endpoints; and (viii) studies without clear treatment or control groups, e.g., treatment not considered digital or CBT, treatment groups involving other interventions, or no proper active/passive controls.

For included articles, any cognitive or behavioral interventions through digital platforms (e.g., internet, mobile, computer-based, VR, …) were considered dCBT groups, and interventions delivered in traditional ways (e.g., face-to-face) were considered active control groups. Groups without active interventions (e.g., placebo, sham, waitlist, …) were considered passive control groups.

### 2.4. Database and Search Strategy

This review included four databases: OVID Medline, Embase, Cochrane Library (Trials), and PsycINFO. The search strategy was set using terms based on the target symptoms (panic disorder or agoraphobia) and intervention type (dCBT). For target symptoms, terms synonymous with panic disorder or agoraphobia were determined (panic, panic disorder, agoraphob*). For the intervention type, free text terms were adopted along with established Medical Subject Headings (MeSH), as using only controlled terms was insufficient to cover the words indicating digital therapy. We identified as many search terms as possible to maximize search sensitivity and the number of initially identified records. The search terms for interventions included internet, cellular phone*, cell phone*, cellphone*, mobile phone*, mobilephone*, computer*, microcomputer*, analog computer*, tablet computer*, laptop computer*, digital computer*, digital, mobile application*, virtual reality, online, on-line, web, website*, www, smartphone*, smart phone*, cyberspace*, cyber space*, personal digital assistant, computer-assisted, computer assisted therapy, Internet-based intervention, virtual reality exposure therapy, digital intervention*, electronic health, virtual care, virtual healthcare, virtual healthcare, and virtual medicine. To include only RCTs and exclude other study designs, the search terms were set based on the SIGN guidelines (Healthcare Improvement Scotland, Edinburgh, United Kingdom). The final keyword was confirmed as a combination of the above search terms, using Boolean operators such as AND, OR, or NEAR. No specific restrictions were applied for each database. The database search was conducted on 15 August 2022.

### 2.5. Study Selection

After initial identification and duplicate removal, Han Jung (HJ) and Ki Won Jang (KWJ) screened the reports independently based on the above eligibility criteria. Conflicts and discrepancies were cleared by discussion, reaching an agreement on the final selection.

### 2.6. Risk of Bias (RoB)

RoB for the selected studies was evaluated independently by HJ and KWJ, who agreed on the final decision via discussion, using Cochrane’s RoB [26] tool (https://methods.cochrane.org/risk-bias-2; accessed on 28 February 2025) with seven domains: (i) randomization, (ii) allocation concealment, (iii) participant/therapist blinding, (iv) assessor blinding, (v) attrition bias, (vi) selective reporting, and (vii) other risks of bias. The risk of bias in each domain for each study was assessed as high, low, or unclear using the Cochrane guidelines.

The raters met before the independent assessment to set the strategies for this review and improve the rater agreement. The participant/therapist blinding domain was applied only to studies with two or more different groups in active treatment. If the study had only one treatment group with passive control, it was automatically considered as a low risk of bias. Otherwise, the domain mainly focused on therapist blinding. For studies with behavioral interventions, clinical interventions by the lead researchers should be carefully performed. As they knew the participants’ allocated conditions, these interventions should have been conducted by delegating the therapy to other researchers who were blind to the allocated condition, ensuring that the therapy was fully structured to prevent subjectivity, or carefully monitoring the intervention process to prevent deviation from the protocol [27]. Only studies applying one or more of these procedures were considered to have a low risk of bias in this domain.

### 2.7. Intervention Components

For any arms that applied dCBT, whether or not the treatment content had the intervention components for testing (interoceptive exposure, inhibitory learning, and personalization) was assessed dichotomously as 0 (not applicable) or 1 (applicable) by the established criteria. HJ and KWJ independently assessed whether the selected studies addressed the three intervention components and reached an agreement.

For interoceptive exposure, the content must be mentioned in the intervention or treatment section. Studies that did not explicitly mention the interoceptive exposure content were still considered applicable if they described similar concepts, such as body sensations. However, any therapeutic content as part of a mindfulness program was not considered interoceptive exposure.

For inhibitory-learning-based intensive exposure, we used the seven cardinal inhibitory learning strategies proposed by Craske et al. (2014): expectancy violation, deepened extinction, reinforced extinction, variability, removing safety behaviors, attentional focus, and affect labeling [16]. To be assessed as following the inhibitory learning principles, studies must have used two or more of these seven strategies. Mere variability in the places of exposure was not considered an inhibitory learning strategy.

Personalization was defined as an application of the optimized therapeutic or exposure content (changes in interventions, settings, conditions, or contexts) to individuals. The therapists or automated programs must have provided the idiosyncratic manipulation based on prior assessment. Random multiple contexts without any prior assessment were not considered personalization. Additionally, merely providing multiple settings (except for the places of exposure) for the same individuals was considered variability of inhibitory learning, not personalization.

### 2.8. Data Extraction

In addition to the intervention components, the following characteristics were extracted from each study/group: whether the format of dCBT was app- or VR-based, existence of a therapist guide, target symptoms, recruitment channels, whether the sample type was patient or general public, diagnostic criteria and existence of diagnostic interviews, study location or country, intervention period, number of therapy sessions, and the existence of conflicts of interest. Information regarding the type of control group (active/passive, waitlist/CBT/in vivo exposure), sample size of treatment and control groups, type of primary outcome selected, and effect size (Hedges’ *g*) and its standard error, were also extracted.

For the therapist guide, “guided” referred to any intervention or support by human therapists, and “unguided” referred to fully automated digital therapy without human interventions. This component can also be a key factor in the treatment efficacy, as patients may be less likely to fully engage in the exposure if they do not receive adequate guidance that ensures the content is sufficiently challenging. For outcomes, the following scales were selected for quantitative synthesis: Panic Disorder Severity Scale (PDSS) [28], Mobility Inventory for Agoraphobia (MI) [29], Agoraphobic Cognitions Questionnaire (ACQ) [30], Fear Questionnaire (FQ) [31], Panic and Agoraphobia Scale (PAS) [32], and Inventory of Agoraphobia (IA) [33]. For studies with multiple candidates for the outcome, the scales most commonly used for the symptom severity of panic or agoraphobia (e.g., PDSS, MI) were prioritized. However, for studies where assessor bias was rated as high, the clinician-rated PDSS was avoided to reduce bias in estimating the effect size. HJ extracted the data, and KWJ and the last author (DR) confirmed the extracted study characteristics.

### 2.9. Effect Size Estimation

The effect sizes were defined as the unbiased estimate (Hedges’ *g*) of the between-group difference in the post-treatment scores of the treatment and control groups. When estimating the effect sizes, the intention-to-treat results were preferred to the completers-only results; however, completers-only results were used when intention-to-treat results were unavailable. When a study had two or more dividable treatment groups, they were considered independent groups, and Hedges’ *g*s were computed for all groups. When a study had two or more control groups of the same type (both passive or both active), they were combined with the weighted average and pooled standard deviation.

### 2.10. Statistical Analyses

A meta-analysis was performed by synthesizing the Hedges’ *g* of each study with a random effects model. In this model, the covariances and heterogeneity estimates across studies (*Q* and *I*^2^) were estimated by the maximum likelihood method. The overall effects and subgroup effects for each clinical factor for passive and active controls were estimated with 95% confidence intervals.

To precisely identify the effects of each factor, we adopted a meta-regression model with the three intervention components included as moderators. Other study characteristics extracted for this review were also included in this model if they could provide more predictive power (i.e., increase the omnibus test statistics [*Q* statistics]). The included factors/characteristics were considered fixed-effect variables if they were categorical and random-effect variables if continuous. Overall, the intervention components and study characteristics were included stepwise until the omnibus test statistics of the whole model became the largest (i.e., the model had the strongest explanatory power). Each clinical factor and covariate’s non-standardized estimate and its 95% confidence interval were computed. To highlight the significance of certain intervention components, a supplementary Bayesian regression model (including the same variables as the frequentist model above) was adopted, which can often generate better results in small sample sizes [34,35]. For this analysis, a fixed effect model with the Akaike Information Criterion (AIC) was adopted with a prior distribution of Beta(1, 1).

To identify publication bias, funnel plots were provided with their asymmetry test (Egger’s test) statistics. The plots and test statistics were reported for the overall effects and meta-regression models for passive/active controls. Trim-and-fill analyses were conducted to adjust publication bias. All processes of meta-analyses were conducted by JASP 0.16.2.0 (https://jasp-stats.org/; accessed on 28 February 2025).

## 3. Results

### 3.1. Selected Studies

**Figure 1:**
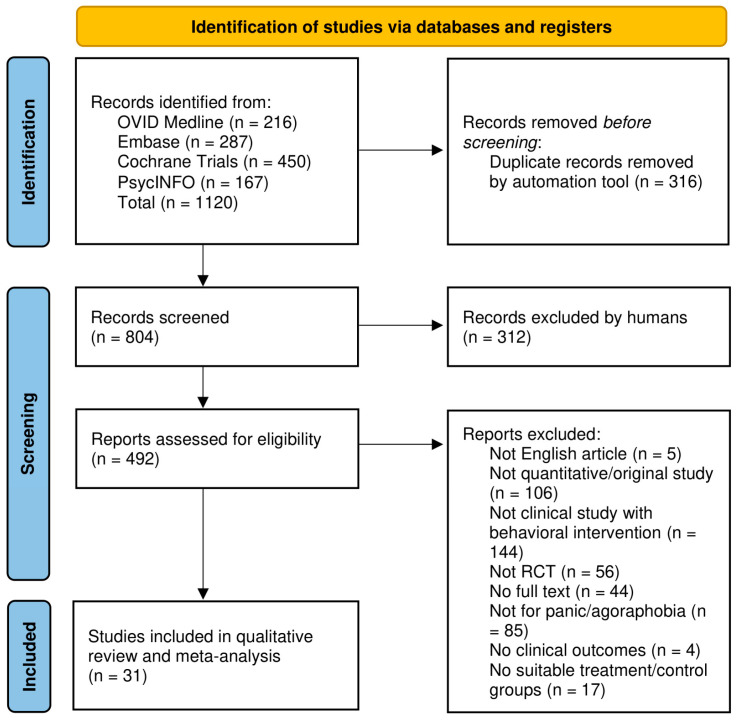
PRISMA flow chart.

Figure 1 presents the PRISMA flow chart [36] illustrating the studies selected for this review. Of the 1120 records identified, 492 reports were assessed in the final selection after de-duplication. The interrater reliability (Gwet’s AC1) [37] between the initial assessments of the two raters was AC1 = 0.92.

Of the assessed reports, 31 studies were selected, comprising 30 journal articles and 1 doctoral dissertation. Specifically, 23 studies, 27 groups, and 1326 participants (669 in treatment groups and 657 in control groups) were included in the passive control condition, and 13 studies, 13 groups, and 663 participants (310 in treatment and 353 in control groups) in the active control condition. A summary of excluded studies is outlined in Figure 1. For the full list of excluded studies, see Supplementary Table S1.

### 3.2. Risk of Bias Assessment

**Figure 2:**
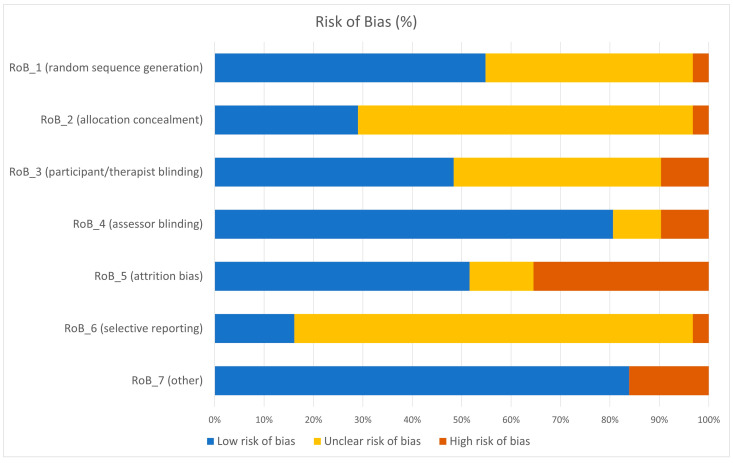
Risk of bias graph.

Figure 2 indicates the overall RoB rates for each risk of bias domain. The initial interrater reliability between the two raters for the first six RoB domains was AC1 = 0.93, 0.68, 0.31, 0.30, 0.24, and 0.85, respectively. Overall, many studies were vulnerable to therapist and attrition biases. Many studies did not report whether therapists were blind to the participant allocation and did not structure the treatment protocol or adequately monitor the process. Additionally, some studies had high attrition rates or uneven distribution of attrition rates across groups. Although many studies successfully corrected bias through the intention-to-treat paradigm, attrition bias may have caused an overestimation of clinical effects. For the assessment of individual studies and the reasoning, see Supplementary Table S2.

### 3.3. Assessment of Intervention Components

Of the 31 selected studies, 25 were considered to involve interoceptive exposure in their dCBT content, and the other 6 were not. Four studies were considered to have inhibitory-learning-based principles or strategies, and five studies were considered to include personalized content. The interrater reliability for interoceptive exposure, inhibitory learning, and personalization in the initial assessment was AC1 = 0.67, 0.79, and 0.84, respectively. For clinical factor ratings of each study and the reasoning, see Supplementary Table S2.

### 3.4. Study Characteristics

Table 1 lists the extracted characteristics for the selected studies or groups. Most dCBTs were performed using computer or mobile applications in non-VR environments. Most studies included therapist guidance or support. Study locations were mostly Europe or Oceania, followed by Asia, North America, and Europe and Oceania combined. The intervention periods ranged from 4 to 12 weeks. The number of sessions of dCBT ranged from 4 to 16, except for one daily chatbot study. Ten of the thirty-one studies declared conflicts of interest.

### 3.5. Overall and Subgroup Effects

**Figure 3:**
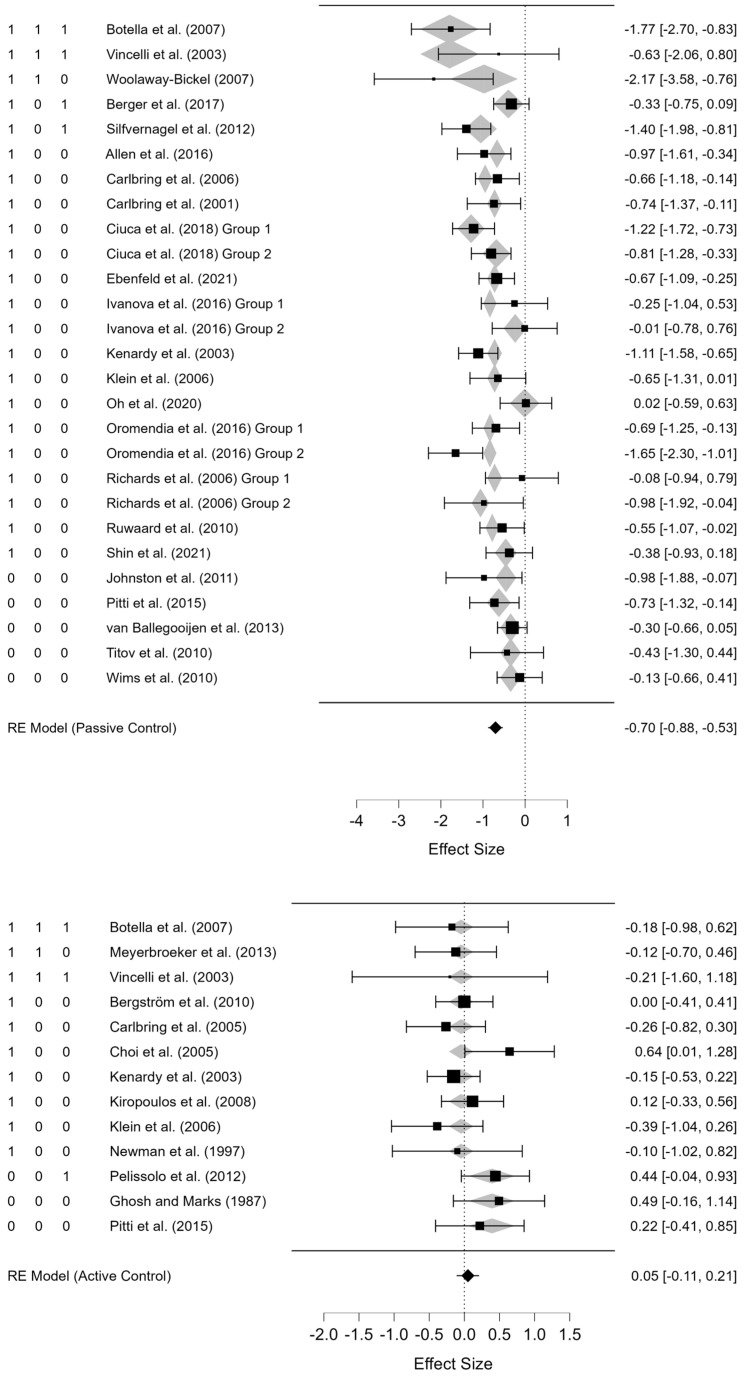
Forest plots for passive (**above**) and active (**below**) controls for the included studies [38,39,40,41,42,43,44,45,46,47,48,49,50,51,52,53,54,55,56,57,58,59,60,61,62,63,64,65,66,67,68]. Note: The numbers on the left refer to interoceptive exposure, inhibitory-learning-based exposure, and personalization, respectively, coded as 1 (present) or 0 (absent). Lower scores (left) favor dCBT and higher scores (right) favor control. The gray diamonds indicate the effect sizes estimated by the meta-regression models.

Table 2 shows the overall and subgroup effects for passive and active controls. Overall, the effect sizes of dCBT were significantly higher than passive controls and not significantly different from active controls. Also, a considerable heterogeneity of effect sizes was observed across studies for passive control, *Q*(26) = 57.71, *p* < 0.001, *I*^2^ = 52.65%, indicating considerable heterogeneity in the clinical efficacy across studies. Regarding active control, no significant heterogeneity was observed, *Q*(12) = 12.97, *p* = 0.371, *I*^2^ = 1.01%. Figure 3 illustrates forest plots describing the individual studies’ effect sizes and confidence intervals for passive and active controls.

For subgroup effects, dCBTs including the hypothesized intervention components tended to have stronger clinical effects, especially with passive controls, although statistical testing was not performed to avoid Type I errors from multiple comparisons. As hypothesized, dCBTs with interoceptive exposure, inhibitory learning, or personalization components tended to have stronger clinical efficacy compared to dCBTs without such components. However, personalized dCBTs showed lower efficacy against active controls compared to non-personalized dCBTs. This may have derived from the relatively small sample size of the dCBT against active control groups, and further studies will be needed to identify the results more accurately.

Also, despite some numerical differences in effect sizes between subgroups, considerable asymmetry in sample sizes was observed. Since all subgroups included at least three studies for both controls, meta-regression analyses seemed appropriate; however, the results should be interpreted with caution because data with uneven covariate distribution may generate invalid results [69].

### 3.6. Meta-Regression

Table 3 (above) shows the final model for passive control resulting from the stepwise procedure. The model indicated a good fit without heterogeneity; *Q*(5) = 31.82, *p* < 0.001 for the omnibus test and *Q*(21) = 25.89, *p* = 0.211, *I*^2^ = 0.00% for residual heterogeneity. The hypothesized intervention components were all included in this model. Additionally, therapist guidance and the number of sessions were included. Interoceptive exposure, therapist guidance, and the number of sessions showed significant confidence intervals, but inhibitory learning and personalization did not, although the stepwise procedure recommended their inclusion. The insignificant results of inhibitory learning and personalization in traditional statistics may have resulted from their small sample sizes. The current confidence intervals show limited information about whether dCBTs with inhibitory learning or personalization may lead to better efficacy; therefore, a supplementary analysis will be helpful for drawing conclusions.

The supplementary Bayesian analysis suggested the inclusion of inhibitory learning and personalization in the model. The Bayes factors for all predictors, including inhibitory learning and personalization, indicated moderate or high evidence for inclusion; therefore, inhibitory learning and personalization may also increase the clinical effects. The effect sizes predicted by the meta-regression model for the individual studies are indicated as gray diamonds in Figure 3.

Table 3 (below) represents the final model for active control deduced by the stepwise procedure. The final model indicated a good fit without heterogeneity; *Q*(1) = 5.31, *p* = 0.021 for the omnibus test and *Q*(11) = 7.66, *p* = 0.743, *I*^2^ = 0.00% for heterogeneity. Unlike passive control, only interoceptive exposure was included in the model, and the Bayes factor indicated strong evidence for the inclusion of this factor. Therefore, inhibitory learning and personalization did not affect the clinical efficacy of dCBT against active control. Unlike interoceptive exposure, the effects of inhibitory learning and personalization have to be confirmed through further studies, considering the small sample sizes included in the current review.

### 3.7. Publication Bias

**Figure 4:**
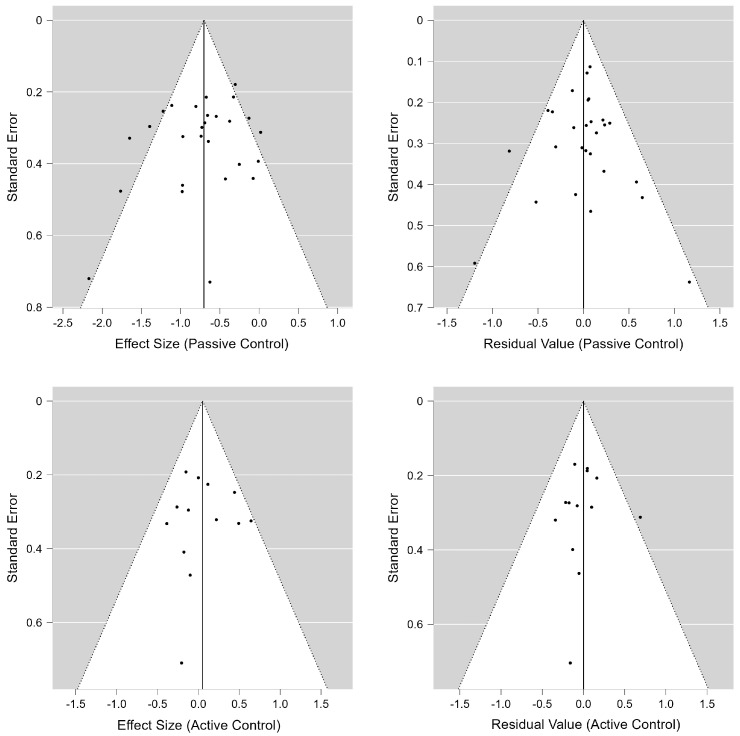
Funnel plots for overall effects and meta-regression.

Figure 4 indicates the funnel plots describing the overall effects and meta-regression models for passive and active controls. Overall, no publication bias was observed through visual inspections or statistical analyses. For passive controls, Egger’s tests were insignificant for the overall effect (*z* = −1.268, *p* = 0.205) and the meta-regression (*z* = 0.078, *p* = 0.938) models, and the trim-and-fill analysis did not result in any changes to the original results. For active controls, Egger’s tests were insignificant for the overall effect (*z* = 0.023, *p* = 0.982) and meta-regression (*z* = −0.218, *p* = 0.827), and the trim-and-fill analysis did not change the results.

## 4. Discussion

This review extends prior knowledge about digital therapy, not only confirming the clinical efficacy of dCBT but also suggesting intervention components to improve its effectiveness for panic disorder and agoraphobia. This review used the largest sample size to date among the quantitative reviews examining digital therapies for panic disorder and agoraphobia. Overall, it seems that dCBT is effective and equivalent to face-to-face CBT despite heterogeneity reported across studies. Notably, the ostensibly varied effect sizes were explained solely by intervention effects, without any external attributes such as population effects. The clinical components implemented differently across studies, such as interoceptive exposure, inhibitory learning principle, or personalization, accounted for the observed heterogeneity. As such, adopting these clinical components or therapist guidance could improve the efficacy of dCBT for panic disorder and agoraphobia.

The current review confirms the clinical efficacy of dCBT for anxiety disorders, in line with previous studies [9,10,12]. Notably, our findings suggest that the variability in dCBT outcomes across studies may be explained, at least in part, by specific intervention components. This represents a novel insight not highlighted in earlier research, underscoring the importance of considering these components when evaluating dCBT for panic disorder and agoraphobia.

Specifically, this review concluded that the following intervention components may improve dCBT’s effectiveness. First, interoceptive exposure significantly increased the clinical effects in passive and active controls. This finding extends Pompoli et al.’s (2018) results on general CBT to the digital field [12]. As interoceptive exposure is indispensable for panic and agoraphobia, digital therapies are encouraged to adopt related content, including programs that have difficulty combining interoceptive components such as VR.

Regarding inhibitory-learning-based exposure, a considerable increase in treatment effect was identified with passive controls. This is the first meta-analytic review to determine the effect of inhibitory learning for panic disorder and agoraphobia. Although the number of studies included was limited, future digital therapies for panic disorder and agoraphobia may consider applying inhibitory learning, as its effectiveness has been recognized in clinical fields on robust theoretical bases [16,70]. Digital environments are particularly suitable for incorporating inhibitory learning strategies automatically, for example, varying stimuli, situations, or designs [71].

The clinical significance of personalization is germane to the advancement of psychotherapy, represented by precision medicine and a transdiagnostic approach [18,20]. Recently, personalized virtual reality has shown high potential to serve as an effective exposure environment for patients with panic disorder [72]. Personalization was included among the components to increase dCBT’s effects for panic disorder and agoraphobia in the passive control model, but not in the active control. This is roughly consistent with previous meta-analysis [24]; personalized or tailored CBT may increase clinical effects, but further exploration is required to draw more reliable conclusions.

Despite the significance of interoceptive exposure, inhibitory learning, and personalization, this review identified that these elements are not yet widely used in dCBTs. Instead, most of the dCBT programs relied on simple therapeutic techniques such as repeated exposure or the introduction of imaginal exposure, and most assigned the same fixed content to all participants. Additionally, dCBTs should pay more attention to establishing evidence-based principles and optimizing treatment protocols. In particular, we suggest that clinicians consider applying the three clinical components covered in this review to maximize the efficacy of dCBT for panic disorder and agoraphobia.

Moreover, therapist guidance or support also increased the effect, although analyzed post hoc. This result is consistent with previous reviews and meta-analyses indicating its better clinical outcomes and adherence compared to unguided CBTs [73,74,75,76]. Although many recent digital therapies have pursued automated treatment based on machine learning or artificial intelligence [77,78], allowing more room for therapists will relieve the rigidity of inflexible, algorithm-based programs. Governments and policymakers should carefully consider therapists’ roles when adopting dCBTs as a replacement for conventional therapies.

These findings may have important implications for clinical practice, training, and patient outcomes. Although further confirmation may be needed, incorporating interoceptive exposure, inhibitory learning, and personalization into routine dCBT protocols can enhance treatment effectiveness and guide more precise, individualized care. Training programs should emphasize these key components to equip clinicians with the necessary skills, ensuring better patient engagement and symptom relief. By applying these insights in practice, policymakers and healthcare providers can optimize dCBT interventions as a reliable and scalable treatment option for panic disorder and agoraphobia.

### 4.1. Limitations

Although this review presented answers to address previous uncertainties, the high therapist and attrition biases and conflicts of interest in several studies suggest that the estimated effect sizes should be considered with caution. Although including treatment components such as interoceptive exposure, inhibitory learning, or personalization removed the numerical heterogeneity values, the treatment efficacy can also depend on other factors such as patient features (severity of symptoms, clinical stages, etc.) or implementation features (therapist characteristics, environmental/random effects, etc.). Additionally, methodological issues within this review need to be acknowledged. Researchers argue that conclusions based on subgroup analyses are being misused without methodological rigor [79,80]. These criticisms include the improper application of post hoc rather than a priori analyses conducted outside the randomization control, which leads to inconsistencies with previous results [79,81].

In this context, the limitations of this review include the intervention components being retrospectively and only qualitatively assessed by the researchers with certain hypotheses. As the effect sizes in this review reflect the study effects irrespective of the random allocation of the study design, the results may have been compounded by variables unrelated to pure treatment effects. Another issue is that this review only appraised the study quality with a risk of bias tool without scoring the overall quality with tools like Jadad and incorporating it in the data synthesis. Moreover, although this review included a sufficient number of studies overall, the small sample sizes within some subgroups hindered the securing of sufficient statistical power or offsetting confounders.

### 4.2. Conclusions and Implications

This review highlights the need for a more evidence-based, organized approach that effectively leverages the strengths of dCBT to optimize treatment. Interoceptive exposure, inhibitory learning, and personalization are promising elements that could be applied to dCBT for panic disorder and agoraphobia. Clinicians may benefit from incorporating these important treatment principles into dCBT protocols to enhance treatment outcomes and optimize dCBT interventions for panic disorder and agoraphobia.

## Figures and Tables

**Table 1:** Study characteristics.

Article	Format	Guide	Symptom	Recruitment	Sample	Diagnosis ^i^	Country	Period	#Sessions	COI	Control	Control Type	*n* Treat.	*n* Cont.	Outcome
Allen et al. (2016) [38]	App	Guided	Panic	Online	Public	MINI	AU	8 weeks	5	No	Passive	Waitlist	16	31 ^ii^	PDSS-SR
Berger et al. (2017) [39]	App	Unguided	PD/A	Clinic	Patient	SCID	CH/DE	9 weeks	6	Yes	Passive	Waitlist	48	41	MI-Alone
Bergström et al. (2010) [40]	App	Guided	PD/A	Clinic	Patient	MINI	SE	10 weeks	10	No	Active	CBT	44	49 ^ii^	PDSS
Botella et al. (2007) [41]	VR	Guided	PD/A	Clinic	Patient	ADIS	ES	9 weeks	9	Yes	Active	IVE	12	12	PDSS
											Passive	Waitlist	12	13	
Carlbring et al. (2006) [42]	App	Guided	Panic	Community/Online	Public	SCID	SE	10 weeks	10	No	Passive	Waitlist	30	30	MI-Alone
Carlbring et al. (2005) [43]	App	Guided	Panic	Community/Online	Public	SCID	SE	10 weeks	10	No	Active	CBT	25	24	MI-Alone
Carlbring et al. (2001) [44]	App	Guided	Panic	Community/Online	Public	CIDI	SE	7–12 weeks	6	No	Passive	Waitlist	21	20	MI-Alone
Choi et al. (2005) [45]	FtF + VR	Guided	PDA	Clinic	Patient	DSM-IV	KR	4 weeks	4	No	Active	CBT	20	20	ACQ
Ciuca et al. (2018) [46] Group 1	App	Guided	Panic	Community/Online	Public	PDSS-SR	RO	12 weeks	16	Yes	Passive	Waitlist	36	38	PDSS-SR
Ciuca et al. (2018) [46] Group 2	App	Unguided	Panic	Community/Online	Public	PDSS-SR	RO	12 weeks	16	Yes	Passive	Waitlist	37	38	PDSS-SR
Ebenfeld et al. (2021) [47]	App	Guided	PD/A	Community/Online	Public	PAS	DE	6 weeks	6	Yes	Passive	Waitlist	45	47	PAS
Ghosh and Marks (1987) [48]	App	Unguided	Agoraph-obia	Clinic	Patient	DSM-III	UK	8 weeks	8	No	Active	CBT	15	25 ^ii,iii^	FQ-Agoraphobia
Ivanova et al. (2016) [49] Group 1	App	Guided	Panic	Community/Online	Public	SCID	SE	10 weeks	8	Yes	Passive	Waitlist	13	12	PDSS-SR
Ivanova et al. (2016) [49] Group 2	App	Unguided	Panic	Community/Online	Public	SCID	SE	10 weeks	8	Yes	Passive	Waitlist	14	12	PDSS-SR
Johnston et al. (2011) [50]	App	Guided	PD/A	Online	Public	MINI	AU	10 weeks	8	No	Passive	Waitlist	20 ^iv^	7	PDSS-SR
Kenardy et al. (2003) [51]	App	Guided	Panic	Clinic/ Community	Patient + Public	SCID	UK/AU	6 weeks	6	No	Active	CBT	41	81 ^v^	MI-Alone
											Passive	Waitlist	41	41	
Kiropoulos et al. (2008) [52]	App	Guided	PD/A	Community/Online	Public	ADIS	AU	6–8 weeks	6	No	Active	CBT	45	35 ^ii^	PDSS
Klein et al. (2006) [53]	App	Guided	Panic	Community/Online	Public	ADIS	AU	6–8 weeks	6	No	Active	CBT	19	18	ACQ
											Passive	Waitlist	19	18	
Meyerbroeker et al. (2013) [54]	VR	Guided	PDA	Clinic	Patient	SCID	NL	8–10 weeks	10	No	Active	IVE	24	22 ^ii^	MI-Alone
Newman et al. (1997) [55]	App	Guided	Panic	Community	Public	SCID (DSM-III)	US	12 weeks	4	No	Active	CBT	9	9 ^ii^	MI-Alone
Oh et al. (2020) [56]	App	Unguided	Panic	Clinic	Patient	MINI	KR	4 weeks	N/A ^vi^	No	Passive	Self-help	21	20 ^ii^	PDSS
Oromendia et al. (2016) [57] Group 1	App	Guided	Panic	Online	Public	MINI	ES	8 weeks	8	Yes	Passive	Waitlist	27	25	PDSS-SR
Oromendia et al. (2016) [57] Group 2	App	Guided	Panic	Online	Public	MINI	ES	8 weeks	8	Yes	Passive	Waitlist	25	25	PDSS-SR
Pelissolo et al. (2012) [58]	VR	Guided	PDA	Clinic	Patient	MINI	FR/LU	12 weeks	12	No	Active	CBT	33	34 ^ii^	PDSS
Pitti et al. (2015) [59]	FtF + VR ^vii^	Guided	Agoraph-obia	Clinic	Patient	CIDI	ES	11 weeks	11	No	Active	CBT	19	20 ^ii^	IA
											Passive	Waitlist	19	32 ^ii^	
Richards et al. (2006) [60] Group 1	App	Guided	Panic	Community/Online	Public	ADIS	AU	6–8 weeks	6	No	Passive	Waitlist	12	9	ACQ
Richards et al. (2006) [60] Group 2	App	Guided	Panic	Community/Online	Public	ADIS	AU	6–8 weeks	6 + 6	No	Passive	Waitlist	11	9	ACQ
Ruwaard et al. (2010) [61]	App	Guided	Panic	Community	Public	DSM-IV	NL	11 weeks	7	Yes	Passive	Waitlist	27	31	PDSS-SR
Shin et al. (2021) [62]	VR	Unguided	Panic	Clinic	Patient	MINI (DSM-V)	KR	4 weeks	12	No	Passive	Waitlist	33	21	PDSS
Silfvernagel et al. (2012) [63]	App	Guided	Panic	Online	Public	SCID	SE	8 weeks	6–8	No	Passive	Waitlist	29	28 ^viii^	PDSS
Titov et al. (2010) [64]	App	Guided	PD/A	Online	Public	MINI	AU	6–8 weeks	6	No	Passive	Waitlist	10	11	PDSS-SR
van Ballegooijen et al. (2013) [65]	App	Guided	Panic	Community/Online	Public	PDSS-SR	NL	6–12 weeks	6	No	Passive	Waitlist	63	63	PDSS-SR
Vincelli et al. (2003) [66]	FtF + VR	Guided	PDA ^ix^	Clinic	Patient	DSM-IV	IT	8 weeks	8 + 1	No	Active	CBT	4	4	FQ
											Passive	Waitlist	4	4	
Wims et al. (2010) [67]	App	Guided	PD/A	Online	Public	MINI	AU	6–8 weeks	6	No	Passive	Waitlist	29	25	MI-Alone
Woolaway-Bickel (2007) [68]	App	Unguided	Panic	Community	Public	ADIS	US	10 weeks	10	No	Passive	Waitlist	7	6 ^ii,x^	Agoraphobic Symptom Composite (FQ + MI)

^i^ Diagnostic interviews adopted DSM-IV criteria unless otherwise specified. ^ii^ Completers only. ^iii^ Therapist + book groups combined. ^iv^ Clinician-supported + coach-supported groups combined. ^v^ CBT6 + CBT12 combined. ^vi^ Daily chatbot study. For statistical analysis, the number of weeks (4) was used in place of the number of sessions. ^vii^ Includes medication of antidepressant (paroxetine) along with face-to-face CBT. ^viii^ Includes six patients (three for each group) not diagnosed with panic disorder or agoraphobia. ^ix^ Although the treatment content targeted panic disorder and agoraphobia, the inclusion criteria incorporated any patients with DSM-IV anxiety disorders. ^x^ As the number of completers for each group was not reported, each group’s sample size was derived from the total number of completers divided by the number of groups. Abbreviations: VR, virtual reality; FtF, face-to-face; PD/A, panic disorder with or without agoraphobia; PDA, panic disorder with agoraphobia; DSM, Diagnostic and Statistical Manual of Mental Disorders; MINI, Mini-International Neuropsychiatric Interview; SCID, The Structured Clinical Interview for DSM; ADIS, Anxiety and Related Disorders Interview Schedule; CIDI, Composite International Diagnostic Interview; AU, Australia; CH, Switzerland; DE, Germany; SE, Sweden; ES, Spain; KR, South Korea; RO, Romania; UK, United Kingdom; NL, Netherlands; US, United States; FR, France; LU, Luxembourg; IT, Italy; #Sessions, number of sessions; COI, conflict of interest; CBT, cognitive behavioral therapy; IVE, in vivo exposure; PDSS/PDSS-SR, Panic Disorder Severity Scale/Panic Disorder Severity Scale-Self Report; MI, Mobility Inventory; ACQ, Agoraphobic Cognitions Questionnaire; PAS, Panic and Agoraphobia Scale; FQ, Fear Questionnaire; IA, Inventory of Agoraphobia.

**Table 2:** Overall and subgroup effects.

		Passive Control					Active Control				
		*k*	*m*	*n*	*g* [95% CI]	*I* ^2^	*k*	*m*	*n*	*g* [95% CI]	*I* ^2^
Overall		23	27	1326	−0.70 [−0.88, −0.53]	53	13	13	663	0.05 [−0.11, 0.21]	1
ite	1	18	22	1047	−0.76 [−0.96, −0.57]	53	10	10	517	−0.05 [−0.22, 0.13]	0
	0	5	5	279	−0.40 [−0.64, −0.16]	0	3	3	146	0.39 [0.06, 0.72]	0
ile	1	3	3	46	−1.60 [−2.28, −0.92]	0	3	3	78	−0.15 [−0.59, 0.30]	0
	0	20	24	1280	−0.66 [−0.83, −0.49]	50	10	10	585	0.08 [−0.10, 0.26]	12
pe	1	4	4	179	−1.00 [−1.62, −0.38]	65	3	3	99	0.24 [−0.16, 0.63]	0
	0	19	23	1147	−0.66 [−0.83, −0.49]	45	10	10	564	0.02 [−0.15, 0.19]	0

Note: Lower scores favor digital CBT and higher scores favor control. For each subgroup, 1 refers to the inclusion of each clinical characteristic (ite, ile, pe) and 0 refers to the absence. *I*^2^ statistics were described in percentages. Abbreviations: ite, interoceptive exposure; ile, inhibitory-learning-based intensive exposure; pe, personalization; *I*^2^, proportion of study variance due to heterogeneity; *k*, number of studies; *m*, number of groups; *n*, number of total participants; CI, confidence interval.

**Table 3:** Meta-regression models.

**Meta-Regression Model for Passive Control [*R*^2^ = 0.551, *I*^2^ = 0%]**				
**Predictor**	**B**	**SE**	**95% CI**	**BF_inclusion_**
Intercept	0.59	0.26	[0.08, 1.10]	
ite (1)	−0.38	0.15	[−0.67, −0.09]	22.92 **
ile (1)	−0.63	0.38	[−1.37, 0.12]	6.44 *
pe (1)	−0.27	0.20	[−0.66, 0.12]	4.27 *
Guide (guided)	−0.60	0.15	[−0.90, −0.30]	>100 ***
#Sessions	−0.06	0.02	[−0.09, −0.02]	36.07 **
**Meta-Regression Model for Active Control [*R*^2^ = 0.409, *I*^2^ = 0%]**				
**Predictor**	**B**	**SE**	**95% CI**	**BF_inclusion_**
Intercept	0.39	0.17	[0.06, 0.72]	
ite (1)	−0.44	0.19	[−0.81, −0.07]	11.26 **

Note: Lower scores favor stronger effects for digital CBT and higher scores favor stronger effects for control. Maximum likelihood method and Wald test for frequentist models. AIC model selection and Beta(1, 1) prior distribution for Bayesian models. ite, ile, and pe were coded 1 (present) and 0 (absent). Guide was considered dichotomous (guided or unguided) and #Sessions was considered continuous. Abbreviations: ite, interoceptive exposure; ile, inhibitory-learning-based intensive exposure; pe, personalization; Guide, therapist guide; #Sessions, the number of sessions; *R*^2^, coefficient of determination; *I*^2^, proportion of study variance due to heterogeneity; B, unstandardized estimate; SE, standard error; CI, confidence interval; BF_inclusion_, Bayes factor for inclusion in the model. * Moderate evidence for inclusion, ** strong evidence for inclusion, *** extreme evidence for inclusion.

## Data Availability

Data are available upon request to the corresponding authors.

## Supplementary Materials

The following supporting information can be downloaded at https://www.mdpi.com/article/10.3390/jcm14051771/s1, Table S1: Excluded Studies and The Reasoning; Table S2: Risk of Bias and Clinical Factor Assessment.
