# Supplementary material for: Digital Cognitive Behavioral Therapy for Panic Disorder and Agoraphobia: A Meta-Analytic Review of Clinical Components to Maximize Efficacy
Source: J Clin Med. 2025 Mar 6;14(5):1771. doi: 10.3390/jcm14051771 (PMC11900950; doi:10.3390/jcm14051771)
Supplement: Supplementary file 1 [file jcm-14-01771-s001.zip › Table S2. Risk of Bias and Clinical Factors Assessment.pdf]

Article	RoB_1 (random sequence generation)	RoB_2 (allocation concealment)	RoB_3 (participant/therapist blinding)	RoB_4 (assessor blinding)	RoB_5 (attrition bias)	RoB_6 (selective reporting)	RoB_7 (other)	Interoceptive exposure *(ite)*	Inhibitory-learning exposure *(ile)*	Personalization *(pe)*
Allen et al. (2016)	L	L	L	L	H (High dropout rate for the treatment group/did not report complete intention-to-treat results; some participants who had withdrawn during the treatment were excluded from the analysis.)	U	L	1	0	0
Berger et al. (2017)	L	L	L	L	U (Slightly higher dropout rate for the treatment group/no reason for dropout was described.)	U (Available trial protocol exists but was retrospectively registered.)	L	1	0	1 (Tailored and personalized content based on individual responses was applied. The content was primarily based on the

										patients' preferences, but the participants' current symptoms were also considered to adjust the content.)
Bergström et al. (2010)	L	L	U (No explanation as to whether the psychologists involved in the treatment, especially the ones for the group therapy, were blinded.)	L	L	U (Available trial protocol exists but was retrospectively registered.)	L	1	0	0
Botella et al. (2007)	U (No explanation regarding the randomization process.)	U (No explanation regarding the randomization process.)	H (Described that the therapists were blinded until the exposure component was applied, implying that considering the study design, the	U (No explanation as to whether the assessors of PDSS were blind to the treatment condition.)	U (No information regarding the excluded participants.)	U	L	1	1 (Exposure to external and interoceptive stimuli could be conducted simultaneously.) (*Deepened extinction, reinforced extinction.*)	1 (Flexible exposure settings depending on the exposure hierarchy for each patient.)

			therapists should have known the treatment condition in the exposure sessions. This is especially vulnerable to bias for the in vivo exposure (control group).)							
Carlbring et al. (2006)	L	U (Insufficient information regarding the allocation concealment.)	L	L	L	U	L	1	0	0
Carlbring et al. (2005)	L	U (Insufficient information regarding the allocation concealment.)	U (Insufficient information as to whether the therapists were blinded (especially for the control group).)	L	L	U	L	1	0	0
Carlbring et al. (2001)	U (The randomizing process was only described as 'drawing of	U (Insufficient information regarding the allocation concealment.	L	L	L	U	L	1	0	0

	lots.')	)								
Choi et al. (2005)	U (No explanation regarding the randomization process.)	U (No explanation regarding the randomization process.)	U (No explanation as to whether the therapists were blinded (especially for the control group).)	L	U (No explanation regarding attrition.)	U	H (No explanation regarding the VR therapy for this study.)	1	0	0
Ciuca et al. (2018)	L	L	U (Insufficient information as to whether the Skype therapeutic guides were blinded.)	L	H (High overall dropout rate/some reasons for dropout seem to be related to the severity of symptoms or the quality of the treatment.)	L	L	1	0	0
Ebenfeld et al. (2021)	L	U (Insufficient information regarding the allocation concealment.)	L	L	L	L	L	1	0 (The interoceptive exposure session involves content regarding safety behaviors, but the overall content was not considered to contain the	0

									inhibitory learning principle.)	
Ghosh and Marks (1987)	U (No explanation regarding the randomization process.)	U (No explanation regarding the randomization process.)	H (The exposure therapy for the control group was conducted by the first author.)	L	L	U	L	0	0	0
Ivanova et al. (2016)	L	L	U (Insufficient information as to whether the therapists were blinded.)	L	H (Considerable overall dropout rate/higher dropout rate among the treatment groups compared to the waitlist group.)	L	L	1 (As acceptance and commitment therapy, it was considered to include activities equivalent to interoceptive exposure.)	0	0
Johnston et al. (2011)	L	L	L	L	L	U	H (The reported statistical results do not match the descriptive data shown.)	0	0	0
Kenardy et al. (2003)	U (Insufficient information regarding the randomization process.)	U (Insufficient information regarding the randomization process.)	L	L	U (Insufficient information regarding the cause for attrition.)	U	L	1	0	0

Kiropoulos et al. (2008)	L	U (Insufficient information regarding the allocation concealment.)	U (No explanation as to whether the therapists in charge of the face-to-face CBT (control group) were blinded.)	L	L	U	L	1	0	0
Klein et al. (2006)	H (The allocation was performed sequentially, not randomly.)	H (The allocation was performed sequentially, not randomly.)	U (No explanation as to whether the therapists (especially the ones in charge of the control group) were blinded.)	H (The assessor blinding was not kept at the post-assessment.)	L	U	L	1	0	0
Meyerbroeker et al. (2013)	U (No explanation regarding the randomization process.)	U (No explanation regarding the randomization process.)	U (Insufficient information as to whether the therapists were blinded (especially for the exposure in vivo group).)	U (No explanation as to whether the assessors were blinded.)	H (High dropout rate/some reasons for dropout seem to be related to the quality of the treatment.)	H (There is a pre-registered protocol available, but many outcome measures reported in the protocol are missing in the final article.)	L	1	1 (Treatment protocol includes discussion of safety behaviors/crowd density could be manipulated in the VR environments.) (*Removing safety behaviors, variability*.)	0
Newman et al. (1997)	U (No explanation regarding	U (No explanation regarding	U (No explanation as to whether	L	L	U	L	1	0	0

	the randomization process.)	the randomization process.)	the therapists in charge of the face-to-face CBT (control group) were blinded.)							
Oh et al. (2020)	U (No explanation regarding the randomization process.)	U (No explanation regarding the randomization process.)	L	L	L	U	H (The statistical results presented are questionable/ the chatbot's functionality was not described well enough.)	1 (Considering its nature, the chatbot's interoceptive exposure content may not be shown to every patient.)	0	0
Oromendia et al. (2016)	L	L	U (No explanation as to whether the therapists were blinded.)	L	H (High attrition rate/the attrition rate was significantly higher in a specific group.)	U (Available trial protocol exists but was retrospectively registered.)	L	1	0	0
Pelissolo et al. (2012)	U (No explanation regarding the randomization process.)	L	H (The dCBT and control groups were treated by the same pool of therapists, implying that the therapists were not	U (No explanation as to whether the assessors measuring PDSS were blinded.)	H (High attrition rate/insufficient information regarding the cause for attrition/intention-to-treat results were not	U	L	0	0	1

			blind to the treatment condition.)		reported.)					
Pitti et al. (2015)	L	U (Insufficient information regarding the allocation concealment.)	U (No explanation regarding the CBT therapists.)	L	H (No explanation regarding the cause for attrition/intention-to-treat results were not reported despite high attrition rate.)	U	H (Insufficient information regarding the CBT and VR contents used in this study.)	0	0	0
Richards et al. (2006)	U (No explanation regarding the randomization process.)	U (No explanation regarding the randomization process.)	U (Insufficient information as to whether the therapists providing email contact were blinded.)	H (One of the assessors was not blinded.)	L	U	L	1	0	0
Ruwaard et al. (2010)	U (No explanation regarding the randomization process.)	U (No explanation regarding the randomization process.)	L	L	L	U	L	1	0	0
Shin et al. (2021)	L	U (Insufficient information regarding the allocation concealment.)	L	L	H (High dropout rate for the VR treatment group/most reasons for dropout seem to be related to the	U (Available trial protocol exists but was retrospectively registered.)	L	1	0	0

					quality of the treatment.)					
Silfvernagel et al. (2012)	L	L	L	L	H (High dropout rate for the treatment group.)	U (Available trial protocol exists but seems to have been registered in the middle of the study.)	L	1	0	1
Titov et al. (2010)	L	U (Insufficient information regarding the allocation concealment/ the allocation was performed by the first author.)	L	L	L	L	L	0	0	0
van Ballegooijen et al. (2013)	L	U (Insufficient information regarding the allocation concealment.)	L	L	H (High dropout rate/no explanation regarding the cause for attrition.)	L	L	0	0	0
Vincelli et al. (2003)	U (No explanation regarding the randomization process.)	U (No explanation regarding the randomization process.)	U (No explanation as to whether the therapists were blinded (especially for the CBT control group).)	L	L	U	L	1	1 (Participants were reinforced to focus on cognitive assessment during the graded exposure and	1 (The therapists collected the anxiety and distress levels of the patients and were

									encouraged to carry out self-exposure.) (*Reinforced extinction, attentional focus.*)	able to change the settings in VR.)
Wims et al. (2010)	L	U (Insufficient information regarding the allocation concealment.)	L	H (The PDSS assessor was not blinded.)	L	U (Available trial protocol exists but was retrospectively registered.)	L	0	0	0
Woolaway-Bickel (2007)	U (No explanation regarding the randomization process.)	U (No explanation regarding the randomization process.)	L	L	H (High dropout rate/dropout was significantly correlated with the credibility of treatment/intention-to-treat results were not reported.)	U	H (The primary outcome measure was a non-validated, arbitrary composite of FQ and MI.)	1	1 (The exposure therapy focused on fading safety behaviors/the patients were encouraged to do anti-phobic exercises.) (*Removing safety behaviors, reinforced extinction.*)	0
